# CircZBTB44 promotes renal carcinoma progression by stabilizing HK3 mRNA structure

**DOI:** 10.1186/s12943-023-01771-5

**Published:** 2023-04-27

**Authors:** Tushuai Li, Yue Gu, Baocai Xu, Kamil Kuca, Jie Zhang, Wenda Wu

**Affiliations:** 1 School of Food and Biological Engineering, Hefei University of Technology, 420 Feicui Road, Hefei, 230009 China; 2 Wuxi School of Medicine, Jiangnan University, Wuxi, 214013 China; 3 MOE Joint International Research Laboratory of Animal Health and Food Safety, College of Veterinary Medicine, Nanjing Agricultural University, Nanjing, 210095 China; 4 Key Laboratory of Anti-inflammatory and Immune Medicine, Ministry of Education, Institute of Clinical Pharmacology, Anhui Medical University, Hefei, 230032 China; 5 School of Biology and Food Engineering, Changshu Institute of Technology, 99 Southern Sanhuan Road, Suzhou, 215500 China; 6 Department of Chemistry, Faculty of Science, University of Hradec Kralove, Hradec Kralove, 50003 Czech Republic

**Keywords:** circZBTB44, HK3, Renal carcinoma, IGF2BP3, HNRNPC, m6A

## Abstract

CircZBTB44 (hsa_circ_0002484) has been identified to be upregulated in renal cell carcinoma (RCC) tissues, while its role and contribution in RCC remain elusive. We confirmed the overexpression of circZBTB44 in RCC cells compared to normal kidney cell HK-2. CircZBTB44 knockdown suppressed the viability, proliferation, and migration of RCC cells and inhibited tumorigenesis in xenograft mouse models. Heterogeneous Nuclear Ribonucleoprotein C (HNRNPC) and Insulin-like growth factor 2 mRNA-binding protein 3 (IGF2BP3) are two RNA binding proteins of circZBTB44. HNRNPC facilitated the translocation of circZBTB44 from nuclei to cytoplasm via m6A modification, facilitating the interaction of IGF2BP3 and circZBTB44 in the cytoplasm of RCC cells. Furthermore, circZBTB44 upregulated Hexokinase 3 (HK3) expression by binding to IGF2BP3 in RCC cells. HK3 exerted oncogenic effects on RCC cell malignant behaviors and tumor growth. In the co-culture of RCC cells with macrophages, circZBTB44 promoted M2 polarization of macrophages by up-regulating HK3. In summary, HNRNPC mediated circZBTB44 interaction with IGF2BP3 to up-regulate HK3, promoting the proliferation and migration of RCC cells *in vitro *and tumorigenesis *in vivo*. The results of the study shed new light on the targeted therapy of RCC.

**Supplementary Information:**

The online version contains supplementary material available at 10.1186/s12943-023-01771-5.

## Introduction

Renal cell carcinoma (RCC) is the most common type of primary renal tumor, characterized by high mortality, with 179,368 deaths in 2020 globally [1]. RCC accounts for 85% of kidney cancer cases and is prevalent in males and elderly people [2, 3]. Around 70% of RCC cases are clear cell renal cell carcinoma, which is regarded as the most common histological subtype of RCC [4]. Risk factors such as smoking, obesity, hypertension may contribute to RCC development [5]. Despite the treatment options such as radiotherapy, chemotherapy, immunotherapy, and targeted therapy, the incidence of RCC continues to increase, and the prognosis of RCC patients remains unsatisfactory because of treatment resistance and side effects [6, 7]. Thus, it is imperative to elucidate the underlying mechanism of RCC and establish promising therapeutic targets to improve RCC treatment.

Circular RNAs (circRNAs) are a class of noncoding RNAs with single-stranded closed circle structure without the 5’ and 3’ ends and poly (A) tails, making them resistance to degradation and more stable than their linear mRNAs [8]. Substantial evidence indicates that circRNAs are important regulators of gene expression at transcriptional and posttranscriptional levels via modulating gene transcription, sponging microRNAs, binding to RNA-binding proteins (RBPs), or translating into peptides [9–12]. CircRNA dysregulation affects gene expression and contributes to the occurrence and development of various diseases, including cancer [13–15]. For example, in RCC, hsa_circ_0015004 is reported to facilitate RCC proliferation and metastasis by binding to miR-127-3p to regulate the CDKN3/E2F1 axis [16]. Hsa_circ_0000741 accelerates RCC cell growth, migration, and invasion as well as angiogenesis by promoting Phosphatidylethanolamine Binding Protein 1 (PEBP1) ubiquitination and activating the ERK signaling pathway [17]. Hsa_circ_0001741 is revealed to inhibit cell proliferation and migration in clear cell renal cell carcinoma cells by binding to Insulin Like Growth Factor 2 mRNA Binding Protein 2 (IGF2BP2) protein to downregulate serpin peptidase inhibitor clade H, member 1 (SERPINH1) [18]. Based on the analysis of the GSE database, a circRNA derived from the pre-mRNA of ZBTB44, hsa_circ_0002484 (we termed it as circZBTB44), has been identified to be upregulated in the tumor and metastatic tissues of RCC patients [19]. However, the role and contribution of circZBTB44 in RCC remains to be elucidated.

Hexokinases (HKs) are involved in the initial step of glycolysis, and hexokinase 3 (HK3) is one of the HK isoenzymes [20]. It was demonstrated that metabolic reprogramming accompanied with upregulated glycolysis is crucially involved in cancer progression [21]. Glycolysis-associated genes such as HK3, phosphoserine aminotransferase 1 (PSAT1), and ribose 5-phosphate isomerase A (RPIA) are suggested as promising prognostic biomarkers in clear cell RCC. HK3 is reported to be highly expressed in tumor tissues of clear cell RCC patients and is indicated to activate apoptosis and epithelial-mesenchymal transition [22]. Increasing research have indicated that HK3 is an important biomarker in various cancers. For instance, HK3 promotes the survival of acute myeloid leukemia cells via interaction with the B-cell lymphoma-2 Interacting Mediator of cell death (Bim) protein in neutrophil differentiation induced by all-trans retinoic acid [23]. HK3 is closely correlated with the epithelial-mesenchymal transition in colorectal cancer and may play essential roles in the proliferation, survival, and metastases of colorectal cancer [24]. A study also indicates that low expression of HK3 is correlated with immune response and may serve as a biomarker for immunotherapy of non-small cell lung cancer [25]. The prognostic value of HK3 in RCC has been demonstrated in previous studies [22, 26, 27], while its impact on RCC development remains rarely explored.

In this study, we aimed to investigate the role and contribution of circZBTB44 in the progression of RCC. We hypothesized that circZBTB44 promoted RCC development by up-regulating HK3. The findings of this study will provide novel insights into the targeted therapy of RCC patients.

## Materials and methods

### Bioinformatics analysis

The online webserver RBPsuite [28] was used to predict the RBPs binding to circZBTB44. The Human Protein Atlas database [29] was used to reveal the subcellular locations of HNRNPC and IGF2BP3. A sequence-based N6-methyladenosine (m6A) modification site predictor, SRAMP [30], was used to predict the m^6^A modification site of circZBTB44. The *cat*RAPID omics v2.1 [31] was used to predict the mRNAs that bound with IGF2BP3.

### Cell culture

Human RCC cell lines (A498, 769-P, ACHN), renal proximal tubular epithelial cells (HK-2), embryonic kidney 293T cells, and acute monocytic leukemia cell line (THP-1) were provided by American Type Culture Collection (ATCC, USA). The RCC cell line OS-RC-2 was obtained from the National Infrastructure of Cell Line Resource (Beijing, China). HK-2 cells were maintained in Dulbecco’s Modified Eagle’s Medium (DME H-21 4.5 g/Liter Glucose) with 10% fetal calf serum (FBS). A498 cells were incubated in Minimum Essential Medium (MEM Eagles with Earle’s Balanced Salts) containing 10% FBS and 1% non-essential amino acids (NEAA). OS-RC-2, 769-P, and THP-1 cells were cultured in RPMI 1640 (w/o Hepes) medium with 10% FBS. ACHN and 293T cells were cultured in DMEM with 10% FBS. All cells were maintained in humidified atmosphere containing 5% CO_2_ at 37 °C.

### Cell transfection

SiRNAs for circZBTB44 (si-circZBTB44-1/-2/-3), HNRNPC (si-HNRNPC-1/-2/-3), Insulin-like growth factor 2 mRNA-binding protein 3 (IGF2BP3; si-IGF2BP3-1/-2/-3), HK3 (si-HK3-1/-2/-3), and the negative control (si-NC) were provided by GenePharma (Shanghai, China). The pLO5-circZBTB44 overexpression vectors and pLO5-ciR control vectors were provided by GENESEED (Guangzhou, China). The pcDNA3.1/HK3 vectors used for HK3 overexpression were supplied by GenePharma with empty pcDNA3.1 vectors as the negative control. Lipofectamine 3000 reagent (Invitrogen) was used to transfect indicated vectors or plasmids into RCC cells following manufacturer’s protocol.

### qRT-PCR

Total RNA extraction from cells and tumor tissues was performed with TRIzol reagent (Beyotime, Shanghai, China). Complementary DNA was synthesized using a SuperScript III Reverse Transcriptase kit. Then PCR was performed with ChamQ Universal SYBR qPCR Master Mix (Vazyme, China). Relative Transcript levels were analyzed by the 2^−ΔΔCt^ method with GAPDH as the endogenous control. The sequences of primers used in this study are shown below.

### circZBTB44

F: 5’-CCAGGAAGTGAACAAGTCCAA-3’,

R: 5’-GCTGTGGGAAGAGGAGCTAT-3’;

### HNRNPC

F: 5’-GATATTAACCTGGCTGCAGAG-3’,

R: 5’-TGATACACGCTGAGTAGAGG-3’;

### IGF2BP3

F: 5’-CGCCTCATTTACAGTGGGA-3’,

R: 5’-CAGTGTTCACTTGCTCACAG-3’;

### HK3

F: 5’-GATCGAAAGTGACAGCCTG-3’,

R: 5’-CACCTCTAGCACCATCAGG-3’;

### GAPDH

F: 5’-CCTCCTGTTCGACAGTCAG-3’,

R: 5’-CATACGACTGCAAAGACCC-3’.

### RNase R and α-amanitin treatment

RNase R was used to digest ZBTB44, and α-amanitin was used to inhibit the transcription of HK3. Total RNA was cultured with RNase R (3 U/mg, Sigma-Aldrich) for 20 min at 37 °C. For α-amanitin (Medchemexpress, Shanghai, China) treatment, RCC cells were stimulated with 50 mM α-amanitin for 0, 6, 12, 18, and 24 h. The expression levels of circZBTB44, ZBTB44, HK3 and β-actin were assessed by qRT-PCR analysis.

### Fluorescence in situ hybridization (FISH)

The subcellular localization of circZBTB44 was determined by FISH assays using a Fluorescence In Situ Hybridization Kit (RiboBio, Guangdong, China) following the manufacturer’s protocol. Briefly, RCC cells were fixed using 4% paraformaldehyde and treated with 0.5% Triton X-100 (Thermo Fisher). Then cells were hybridized with the specific probes for circZBTB44, followed by counterstaining with DAPI. The images were photographed by a microscope (Nikon, Japan).

### Immunofluorescence staining

The subcellular localizations of HNRNPC and IGF2BP3 were determined by immunofluorescence staining assay. After adhering to the culture slides, cells were washed in PBS, fixed in formaldehyde for 10 min, and blocked in 5% BSA for 10 min. Next, samples were incubated with the Alexa Fluor® 488-labeled primary antibodies against HNRNPC and IGF2BP3. After that, cells were washed in PBS and cultured with the second antibody IgG. Nuclei was stained by DAPI dye.

### Cell viability

A Cell-Counting Kit-8 (CCK-8, Beyotime) was applied to detect the viability of transfected RCC cells. Briefly, RCC cells were plated into 96-well plates at 2000 cells per well and incubated for 1, 2, 3, and 4 days. Next, 10 µl of CCK-8 reagent (Beyotime) was supplemented into each well and cultured for 60 min. Light absorbance of cells was detected with a Multiscan Spectrum (Petenov, Beijing, China) at 450 nm.

### EdU assays

The proliferation potential of RCC cells was assessed by a 5-ethynyl-2’-deoxyuridine (EdU) assay using an EdU Apollo 488 Kit (RiboBio, China). Briefly, RCC cells were grown after indicated transfection in 12-well plates at 10,000 cells per well. EdU was added, and cultured for 2 h, and cells were fixed by 4% paraformaldehyde and dyed with the kit according to manufacturer’s protocol. A fluorescence microscope was applied to capture the images, and the ratio of EdU-positive cells was calculated.

### Transwell assays

The migration of treated RCC cells was measured by Transwell assays using Transwell chambers (Corning, USA). Cells (2 × 10^4^) were grown in the apical layer of the Transwell chambers supplemented with serum free media, and the bottom chambers were added with DMEM with 10% FBS. After culturing for 24 h at 37 °C, the migrated RCC cells were fixed and dyed using 1% crystal violet. Finally, the number of migrated RCC cells was counted in five randomly selected visual fields.

### Wound healing

Transfected RCC cells were grown in 6-well plates and cultured to reach 80% confluence. Then a 20 µL pipette tip was applied to scratch the plates to create a wound. Images at 0 and 12 h were captured using a microscope, and the wound closure was calculated to evaluate the migration of RCC cells.

### Xenograft mouse models

The procedures of animal study were approved by the Ethic Committee of the Jiangnan University (approval number: JNU(SU)2022-0017). BALB/c Nude mice (aged 4 weeks) of both sexes were provided by the Shanghai SLAC Laboratory Animals Co., Ltd. (Shanghai, China). The animals were randomly separated into indicated groups (n = 6 per group). Xenograft mouse models were established by subcutaneously inoculating 1 × 10^7^ transfected ACHN cells into mouse left flank. Mouse tumor volume was recorded using calipers every 3 days since day 7. Four weeks after the inoculation, mice were sacrificed, and tumors were excised and weighed. A body-weight reduction of 20% or more is a parameter for humane endpoint. A combination of general symptoms such as raised fur, hunched back, poor coat conditions, and behavioral changes like lethargy are considered as humane endpoints, after which the animals will be euthanized. Mice were injected intraperitoneally with 3% sodium pentobarbital at a dose of 50 mg/kg (1–1.25 mg per mouse) for anesthesia and were sacrificed by cervical dislocation. Animal death was confirmed by cessation of heartbeat.

### Immunohistochemistry (IHC)

The tumor tissue samples were fixed in formalin, paraffin-embedded, and then sliced into 4 μm sections. After dewaxing, rehydration, and antigen retrieval, the sections were incubated with a specific primary antibody against Ki67 (ab16667, 1/200, abcam) and an HRP-conjugated secondary antibody anti-IgG (ab7090, 1/200, abcam). Images were captured with a microscope.

### RNA pull-down and GST-pulldown

The biotin-labeled circZBTB44 probe (Bio-circZBTB44) and Bio-NC were provided by RiboBio. Briefly, RCC cells were harvested after indicated transfection (2 × 10^7^) and subjected to Radio Immunoprecipitation Assay (RIPA) lysis buffer. Next, cell lysis was cultured with the biotin-labeled probe for 60 min at ambient temperature. Then streptavidin agarose beads (Thermo Fisher) were added to separate the complex. RNA bound to the complex was eluted and the enrichment of HNRNPC and IGF2BP3 was analyzed with Western blot. GST-pulldown assays were applied to assess the interaction of HNRNPC and IGF2BP3. GST-IGF2BP3 or anti-IgG was added into cell lysate of RCC cells and cultured with Glutathione beads (Sigma-Aldrich) for 2 h. The bound proteins in the complex were subject to Western blot analysis.

### RNA immunoprecipitation (RIP) and meRIP

RIP assays were used to investigate the interaction of circZBTB44 and HNRNPC or IGF2BP3 and the interaction of HK3 with IGF2BP3 using a Magna RIP kit (Merck Millipore). Transfected RCC cells were lysed and cultured with magnetic beads conjugated with anti-HNRNPC, anti-IGF2BP3, or anti-IgG at 4℃ overnight with rotation. Next, RNA binding to the indicated proteins was extracted, purified, and subjected to qRT-PCR analysis. For meRIP assays, m6A antibody was used to immunoprecipitate the methylated circZBTB44.

### Co-culture of RCC cells with macrophages

THP-1 cells were treated with 100 ng/ml PMA (Sigma-Aldrich) for 24 h to differentiate into M0 macrophages. Tumor-associated macrophages (TAMs) were obtained after co-culturing with transfected A498 or 769-P cells for 24 h.

### Flow cytometry

The ratio of CD86^+^CD206^−^ and CD206^+^CD86^−^ macrophages was determined using flow cytometry. Cells were fixated with 4% paraformaldehyde at 37 °C for 20 min, and then resuspended in phosphate buffered saline (PBS). Next, cells were cultured with fluorescein 5-isothiocyanate (FITC) conjugated anti-CD206 and CD86-APC antibodies at 4 °C for 60 min. A flow cytometer (FACS) was applied to determine the ratio of CD86^+^CD206^−^ and CD206^+^CD86^−^ macrophages.

### Western blot

Extraction of total protein was conducted using RIPA buffer (Beyotime). After separation with 10% sodium dodecyl sulfate polyacrylamide gel electrophoresis gels, the sample was transferred onto polyvinylidene difluoride membranes, followed by blockage with 5% skim milk. Then the membranes were cultured with the anti-CD86 (ab269587, 1/1000, Abcam), anti-TNF-α (ab183218, 1/1500, Abcam), anti-CD206 (MA5-32498, 1/1000, Thermo Fisher), and anti-ARG-1 (PA5-85267, 1/500, Thermo Fisher) at 4 °C overnight with β-actin as the internal reference. After incubation with the secondary antibodies for 60 min at ambient temperature, an ECL Substrate Kit (Thermo Fisher) was applied to visualize the target protein bands.

### Statistical analysis

GraphPad Prism 8 was applied for data analysis. The results were exhibited as the mean ± standard deviation. Student’s *t*-test was applied to analyze statistical difference between two groups, and one-way analysis of variance was used to analyze the difference among three or more groups. A p value less than 0.05 was considered statistically significant.

## Results

### Expression of circZBTB44 in RCC tissues and cells

**Fig. 1:**
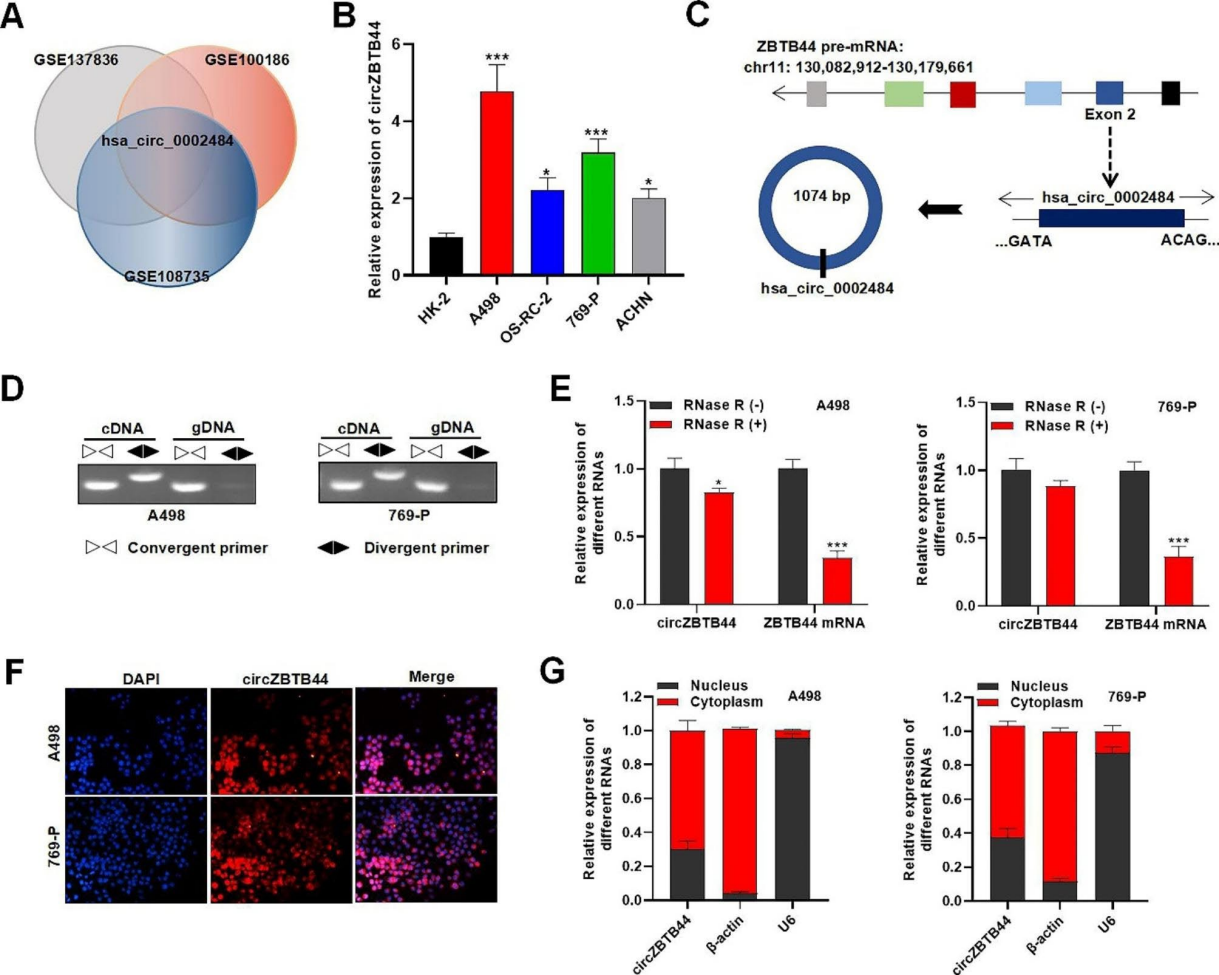
Expression of circZBTB44 in RCC tissues and cells. (**A**) Venn diagram of circZBTB44 (hsa_circ_0002484) upregulated in RCC tumor tissues in GSE100186, GSE108735 and GSE137836. (**B**) qRT-PCR was used to measure the expression of circZBTB44 in RCC cell lines (A498, OS-RC-2, 769-P, ACHN) and human renal proximal tubular epithelial cell line (HK-2). (**C**) The Schematic diagram of genomic mapping and splicing patterns of circZBTB44. (**D**) PCR and electrophoresis analysis was used to detect the existence of circZBTB44 in complementary DNA (cDNA) and genomic DNA (gDNA). (**E**) CircZBTB44 and ZBTB44 mRNA expression in RCC cells after RNase R treatment was detected using qRT-PCR. (**F-G**) FISH and subcellular fractionation assays were performed to detect the subcellular location of circZBTB44 in RCC cells. *P < 0.05, ***P < 0.001

CircZBTB44 (hsa_circ_0002484) has been revealed to be upregulated in RCC tumor tissues based on the analysis of GSE100186, GSE108735, and GSE137836 datasets (Fig. 1A) [19]. We also demonstrated the higher expression of circZBTB44 in RCC cells than that in human renal proximal tubular epithelial cells (HK-2), especially in A498 and 769-P cells (Fig. 1B). CircZBTB44 (chr11:130130750–130,131,824), located on the Chromosome 11, is back-spliced from the host gene ZBTB44 from exon 2 (Fig. 1C). According to the results of PCR and electrophoresis analyses, circZBTB44 was only amplified by divergent primers in cDNA, and no amplification product was observed in gDNA (Fig. 1D). Moreover, we also found that circZBTB44 was resistant to RNase R treatment compared to the ZBTB44 mRNA, suggesting that circZBTB44 was more stable than its linear mRNA due to its circular structure (Fig. 1E). As revealed by FISH and subcellular fractionation assays, circZBTB44 was distributed in both cytoplasm and nuclei of RCC cells, with more circZBTB44 in the cytoplasm (Fig. 1F-G).

### CircZBTB44 promoted RCC cell development in vitro and mouse tumorigenesis in vivo

**Fig. 2:**
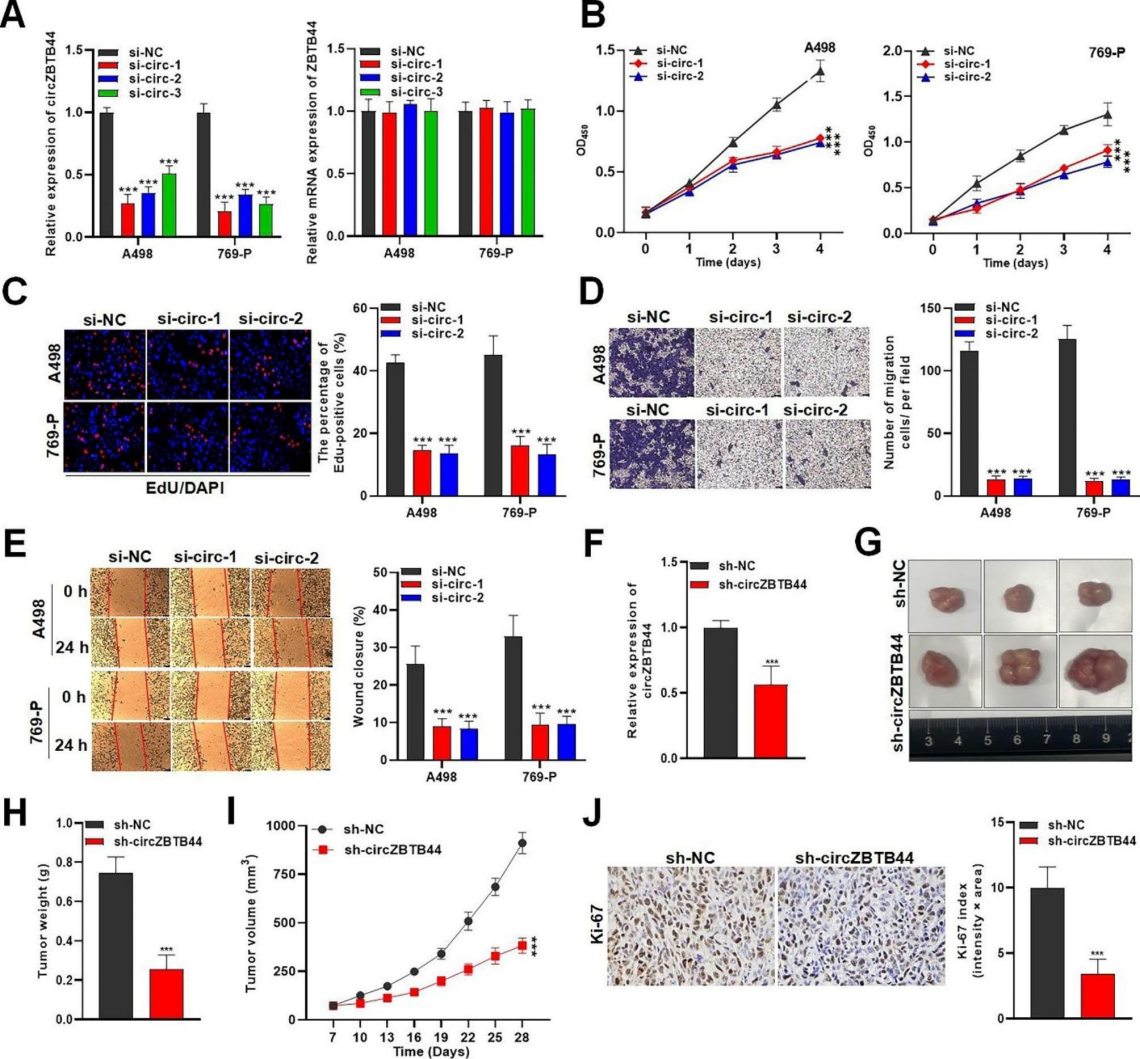
CircZBTB44 promoted RCC cell malignancyin vitro. (**A**) The transfection efficiency of si-circZBTB44-1/-2/-3 as well as the effect of si-circZBTB44-1/-2/-3 on ZBTB44 mRNA expression in RCC cells were analyzed by qRT-PCR. (**B**) CCK-8 and (**C**) EdU assays were used to detect the impact of circZBTB44 knockdown on RCC cell viability and proliferation. (**D**) Transwell and (**E**) wound healing assays were used to detect RCC cell migration after circZBTB44 knockdown. (**F**) qRT-PCR was used to detect the expression of circZBTB44 in xenografts of indicated groups. (**G**) Xenografts from different groups. (**H**) Mouse tumor weight and (**I**) volume in each group. (**J**) IHC assays were performed to detect the protein expression of Ki-67 in mouse tumor tissues. *P < 0.05, ***P < 0.001

The impact of circZBTB44 on RCC cell malignant behaviors was investigated through a series of functional experiments. The expression of circZBTB44 showed significant reduction in RCC cells after transfection of si-circZBTB44-1/-2/-3, and ZBTB44 mRNA expression was not affected by si-circZBTB44. The si-circZBTB44-1 and si-circZBTB44-2 plasmids showed better silencing efficacy and were applied in the following experiments (Fig. 2A). Then we evaluated the viability and proliferation potential of RCC cells, and found that circZBTB44-2 silencing showed evident suppression of RCC cell growth in vitro (Fig. 2B-C). Moreover, the migrated RCC cell number was significantly reduced in the si-circZBTB44-1/-2 group relative to the control (Fig. 2D). The wound healing distance of RCC cells was decreased by circZBTB44 knockdown (Fig. 2E), suggesting that circZBTB44 silencing significantly inhibited RCC cell migratory ability. CircZBTB44 is indicated to act as a cancer promoter in RCC cell growth and migration. Then we examined the effects of circZBTB44 knockdown on mouse tumor growth in vivo. The circZBTB44 levels showed significant decrease in mouse tumor tissue samples in the si-circZBTB44 group relative to the control (Fig. 2F). The tumor size and weight showed evident decrease in the si-circZBTB44 group compared with the control (Fig. 2G-H). Similarly, the growth rate of tumor was reduced after silencing circZBTB44 (Fig. 2I). Furthermore, according to the results of IHC staining, circZBTB44 silencing caused an evident reduction in the expression of Ki-67 protein, suggesting that circZBTB44 stimulated tumor growth in vivo (Fig. 2J).

### CircZBTB44 interacted with HNRNPC and IGF2BP3 in RCC cells

**Fig. 3:**
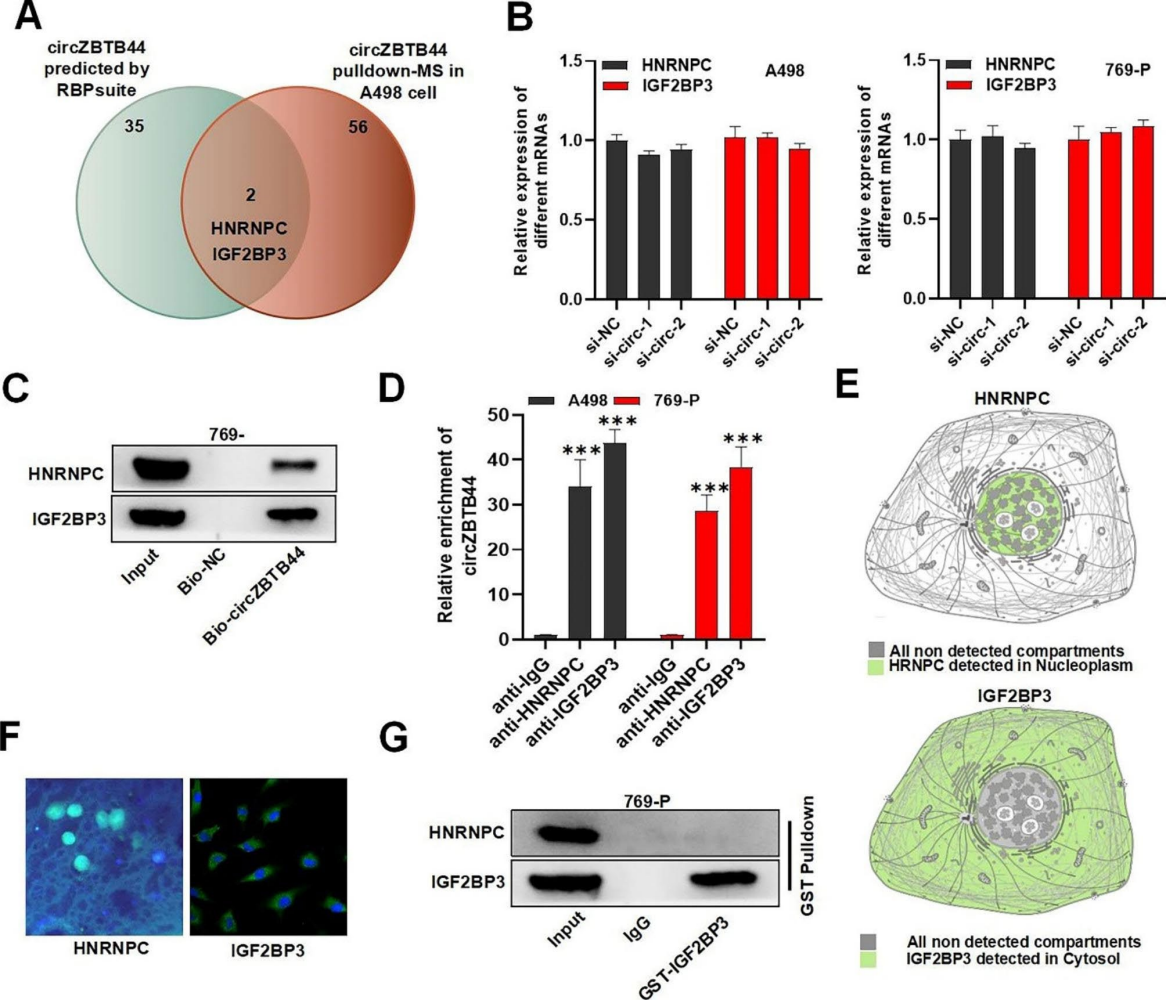
CircZBTB44 interacted with HNRNPC and IGF2BP3 in RCC cells. (**A**) Venn diagram of RNA binding proteins (RBPs) of circZBTB44 based on the prediction on the RBPsuite website and results of RNA pulldown-MS. (**B**) The mRNA levels of HNRNPC and IGF2BP3 in circZBTB44 silenced RCC cells. (**C**) RNA-pulldown assays were performed to detect the interaction of circZBTB44 with HNRNPC or IGF2BP3. (**D**) RIP assays were used to evaluate the binding between circZBTB44 and HNRNPC or IGF2BP3. (**E**) The Human Protein Atlas database was used to predict the subcellular localization of HNRNPC and IGF2BP3. (**F**) The subcellular localizations of HNRNPC and IGF2BP3 were confirmed by immunofluorescence assay. (**G**) GST-pulldown assays were used to examine the interaction between HNRNPC and IGF2BP3. ***P < 0.001

The regulatory mechanism of circZBTB44 in RCC was further explored, based on the prediction on the RBPsuite website and results of RNA pulldown-MS. The results showed that HNRNPC and IGF2BP3 possibly interacted with circZBTB44 (Fig. 3A). Then we examined the impact of circZBTB44 silencing on the expression of HNRNPC and IGF2BP3, and qRT-PCR results indicated that HNRNPC and IGF2BP3 expression were not significantly affected by circZBTB44 silencing in RCC cells (Fig. 3B). RNA-pulldown assays revealed that HNRNPC and IGF2BP3 were abundantly enriched in the complex of bio-circZBTB44, which indicated that circZBTB44 interacted with HNRNPC and IGF2BP3 in RCC cells (Fig. 3C). Similarly, we observed abundant enrichment of circZBTB44 in the precipitates of anti-HNRNPC and anti-IGF2BP3, which verified the interaction between circZBTB44 and HNRNPC or IGF2BP3 (Fig. 3D). The Human Protein Atlas predicted that HNRNPC was mainly distributed in cell nucleus, while IGF2BP3 was primarily located in cell cytoplasm (Fig. 3E), which were further confirmed by immunofluorescence assay (Fig. 3F). We then evaluated the interaction between HNRNPC and IGF2BP3, and the results showed no direct interaction between HNRNPC and IGF2BP3 (Fig. 3G).

### HNRNPC enhanced the interaction of circZBTB44 and IGF2BP3 and mediated circZBTB44 translocation to cytoplasm via m^6^A modification

**Fig. 4:**
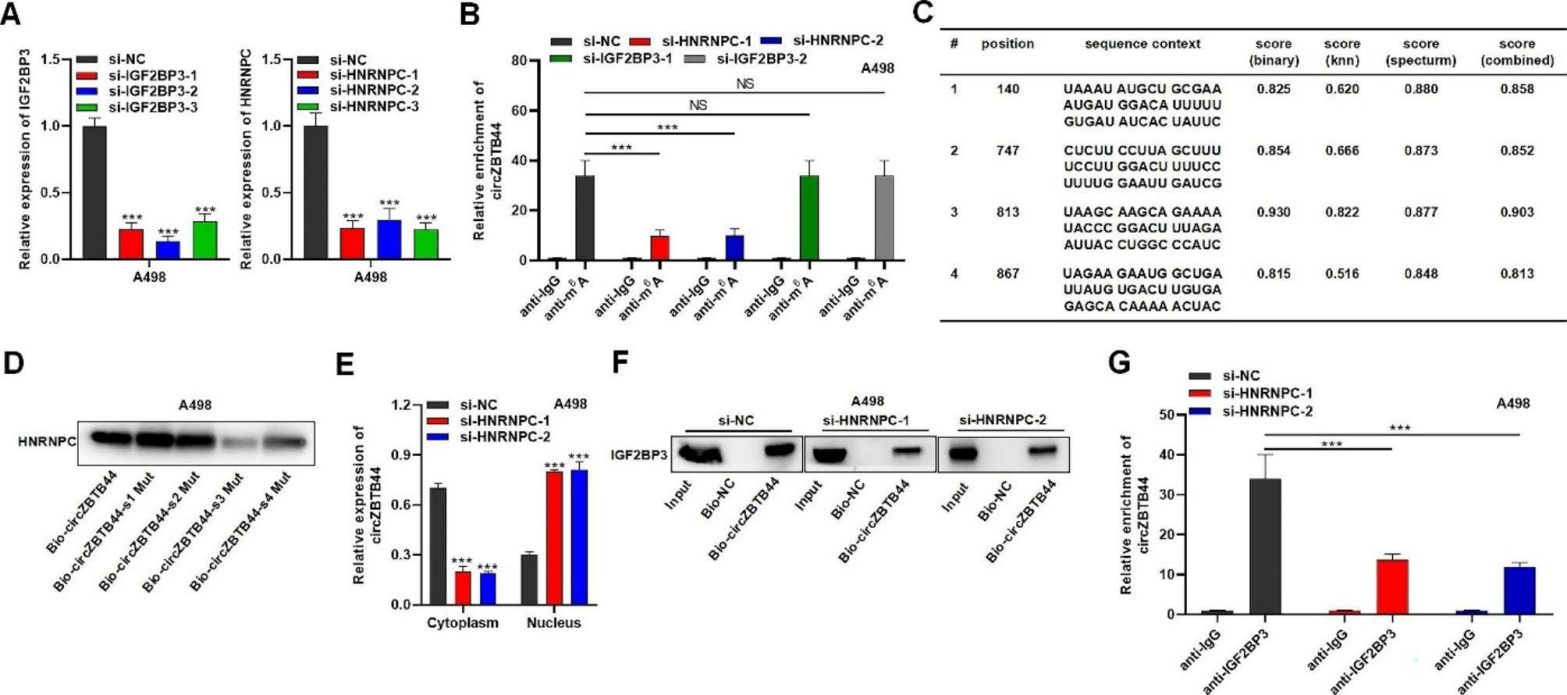
HNRNPC enhanced the interaction of circZBTB44 and IGF2BP3 and mediated circZBTB44 translocation to cytoplasm via m^6^A modification. (**A**) The silencing efficiency of HNRNPC and IGF2BP3 in RCC cells was analyzed by qRT-PCR. (**B**) MeRIP assays were conducted to evaluate the impact of HNRNPC or IGF2BP3 knockdown on circZBTB44 m^6^A modification. (**C**) The m^6^A modification site of circZBTB44 was predicted on the SRAMP database (http://www.cuilab.cn/sramp). (**D**) RNA-pulldown assays were conducted to explore the interaction between HNRNPC and circZBTB44 or mutant circZBTB44. (**E**) qRT-PCR was used to detect the distribution of circZBTB44 in cytoplasm and nucleus of HNRNPC-silenced RCC cells. (**F**) RNA-pulldown assays were applied to investigate the effects of HNRNPC silencing on the binding between circZBTB44 and IGF2BP3 in RCC cells. (**G**) RIP assays were used to evaluate the impact of HNRNPC knockdown on the interaction of circZBTB44 and IGF2BP3 in RCC cells. ***P < 0.001

HNRNPC is a N6-methyladenosine (m^6^A) reader that regulates the RNA processing such as RNA splicing, 3’-end processing, and translation [32, 33]. It was assumed that HNRNPC was involved in the regulation of circZBTB44. qRT-PCR results verified the transfection efficiency of HNRNPC and IGF2BP3 by siRNAs in RCC cells (Fig. 4A). The results of MeRIP assays demonstrated that circZBTB44 was significantly enriched in the precipitates of anti-m^6^A, which was reduced after silencing HNRNPC, and showed no evident change after IGF2BP3 knockdown in RCC cells, suggesting that HNRNPC affected the m^6^A modification of circZBTB44 (Fig. 4B). Moreover, the SRAMP database was used to predict the m^6^A modification site of circZBTB44, and 4 m^6^A sites with very high confidence were found (Fig. 4C). Then the 4 m^6^A sites were mutated, and the results of RNA-pulldown assays indicated that HNRNPC was not enriched in the complex pulled down by Bio-circZBTB44-s3Mut (Fig. 4D). Then we evaluated the expression of circZBTB44 in cytoplasm and nucleus. qRT-PCR indicated that HNRNPC knockdown significantly decreased the cytoplasmic distribution of circZBTB44 and elevated the expression of circZBTB44 in the nucleus of RCC cells (Fig. 4E). The impact of HNRNPC on the interaction of circZBTB44 and IGF2BP3 was further explored, and HNRNPC deficiency evidently decreased the enrichment of IGF2BP3 in the complex of Bio-circZBTB44, suggesting that HNRNPC silencing inhibited the interaction between circZBTB44 and IGF2BP3 in RCC cells (Fig. 4F). Similarly, the results of RIP assays indicated that the enrichment of circZBTB44 showed significant decrease in the precipitates of anti-IGF2BP3 (Fig. 4G). Overall, HNRNPC promoted the translocation of circZBTB44 to the cytoplasm via m6A modification, enhancing the interaction of circZBTB44 and IGF2BP3.

### CircZBTB44 bound with IGF2BP3 to regulate the mRNA stability of HK3

**Fig. 5:**
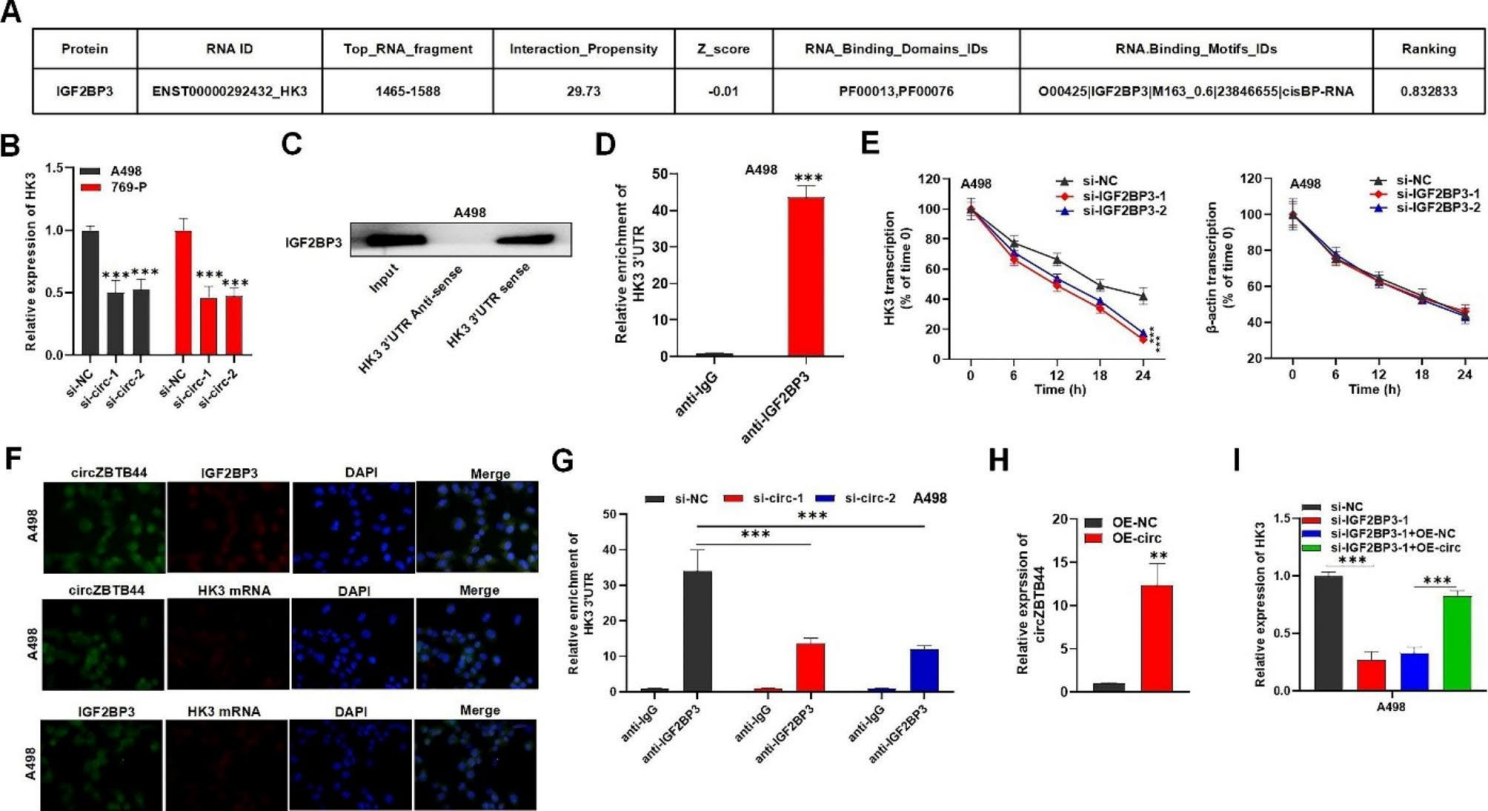
CircZBTB44 interacted with IGF2BP3 to regulate HK3 mRNA stability. (**A**) The catRAPID database (http://service.tartaglialab.com/page/catrapid_group) was used to predict the mRNAs that bound with IGF2BP3. (**B**) The impact of circZBTB44 knockdown on HK3 expression in RCC cells was examined using qRT-PCR analysis. (**C**) The enrichment of IGF2BP3 in the complex of HK3 3’UTR was analyzed using RNA pull-down assays. (**D**) RIP assays were used to verify the interaction of HK3 3’UTR and IGF2BP3 protein in RCC cells. (**E**) The effects of IGF2BP3 silencing on the mRNA stability of HK3 were examined using qRT-PCR in RCC cells treated with α-amanitin. (**F**) FISH assays were conducted to observe the colocalization of circZBTB44, IGF2BP3 and HK3 in RCC cells. (**G**) RIP assays were conducted to evaluate the impact of circZBTB44 knockdown on the interaction of HK3 3’UTR and IGF2BP3 in RCC cells. (**H**) qRT-PCR analysis was used to detect the expression efficiency of circZBTB44 in RCC cells. (**I**) qRT-PCR analysis was used to detect the HK3 expression in RCC cells after indicated transfection. **P < 0.01, ***P < 0.001

IGF2BP3 has been revealed to bind to mRNA and regulate target gene expression [34]. Therefore, the downstream targets of circZBTB44/IGF2BP3 were investigated. According to the results predicted by the catRAPID database and previous studies, we found HK3 most possibly bound with IGF2BP3 and was also upregulated in RCC (Fig. 5A). The impact of circZBTB44 knockdown on HK3 expression was examined, and HK3 was significantly down-regulated in circZBTB44-silenced RCC cells (Fig. 5B). Enrichment of IGF2BP3 was observed in the complex of HK3 3’UTR, which indicated that IGF2BP3 bound to HK3 3’UTR in RCC cells (Fig. 5C). As revealed by RIP assays, HK3 3’UTR was abundantly enriched in the precipitates of anti-IGF2BP3 (Fig. 5D). After α-amanitin treatment, the mRNA stability of HK3 was significantly reduced after silencing IGF2BP3 in RCC cells (Fig. 5E). FISH assays showed the colocalization of circZBTB44, IGF2BP3, and HK3 in the cytoplasm of RCC cells (Fig. 5F). As revealed by RIP assays, the enrichment of HK3 3’UTR in the precipitates of anti-IGF2BP3 exhibited significant reduction in circZBTB44-silenced RCC cells (Fig. 5G). The overexpression efficiency of circZBTB44 was confirmed by qRT-PCR analysis (Fig. 5H). We also observed that HK3 expression was downregulated after IGF2BP3 knockdown and was reversed by overexpressing circZBTB44 in RCC cells (Fig. 5I).

### HK3 promoted RCC growth and metastasis in vitro and in vivo

**Fig. 6:**
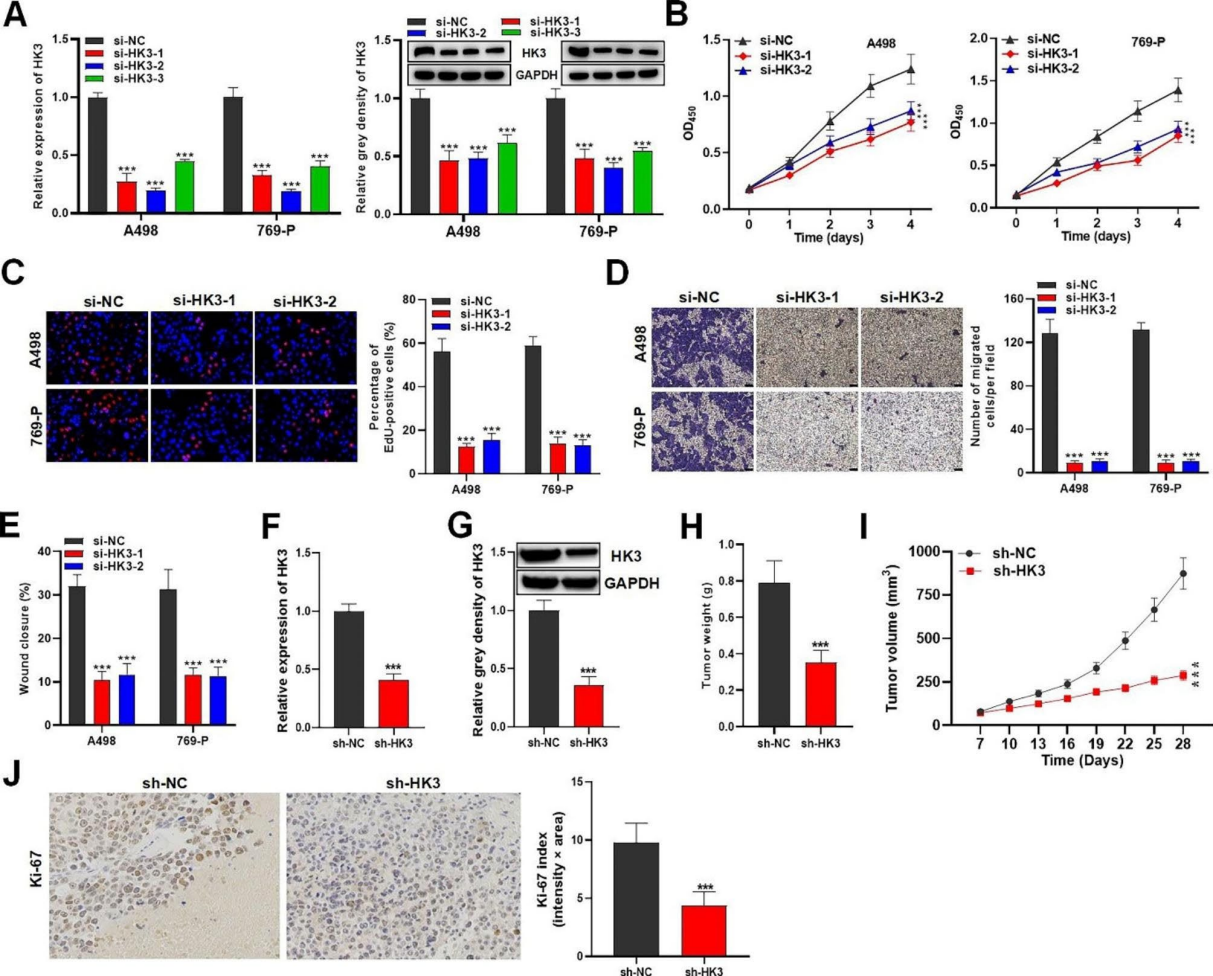
HK3 promoted RCC growth and metastasisin vitro and in vivo. (**A**) qRT-PCR and western blot analyses were used to detect the transfection efficacy of HK3 siRNAs (si-HK3-1/-2/-3) in RCC cells. (**B**) CCK-8 and (**C**) EdU assays were conducted to detect the impact of HK3 knockdown on RCC cell viability and proliferation. (**D**) Transwell and (**E**) Wound healing assays were used to detect the effects of HK3 knockdown on RCC cell migration. (**F-G**) The expression of HK3 in mouse tumor tissues was measured using qRT-PCR and western blot analyses. (**H**) Mouse tumor weight and (**I**) volume in indicated groups. (**J**) IHC assays were conducted to examine the Ki67 protein expression in mouse tumor tissues of indicated groups. ***P < 0.001

Then the effects of HK3 on RCC cell tumorigenesis was investigated. qRT-PCR and western blot analyses revealed the significant reduction of HK3 after transfection of si-HK3-1/-2/-3 in RCC cells (Fig. 6A). HK3 knockdown was demonstrated to significantly inhibit the viability and proliferation ability of RCC cells (Fig. 6B-C). Moreover, RCC cell migratory ability exhibited significant decrease in the si-HK3-1 and si-HK3-2 groups relative to the control (Fig. 6D-E). The impact of HK3 deficiency on RCC tumorigenesis was evaluated using tumor-bearing mouse models. qRT-PCR and western blot analyses showed that HK3 expression was evidently down-regulated in mouse tumor tissues of the sh-HK3 group (Fig. 6F-G). HK3 deficiency induced significant reduction in mouse tumor weight and volume relative to the control group (Fig. 6H-I). Additionally, the Ki67 protein in mouse tumor tissues was revealed to be reduced by HK3 deficiency, suggesting that HK3 knockdown suppressed RCC tumorigenesis in vivo (Fig. 6J).

### CircZBTB44 facilitated RCC growth and metastasis by up-regulating HK3

**Fig. 7:**
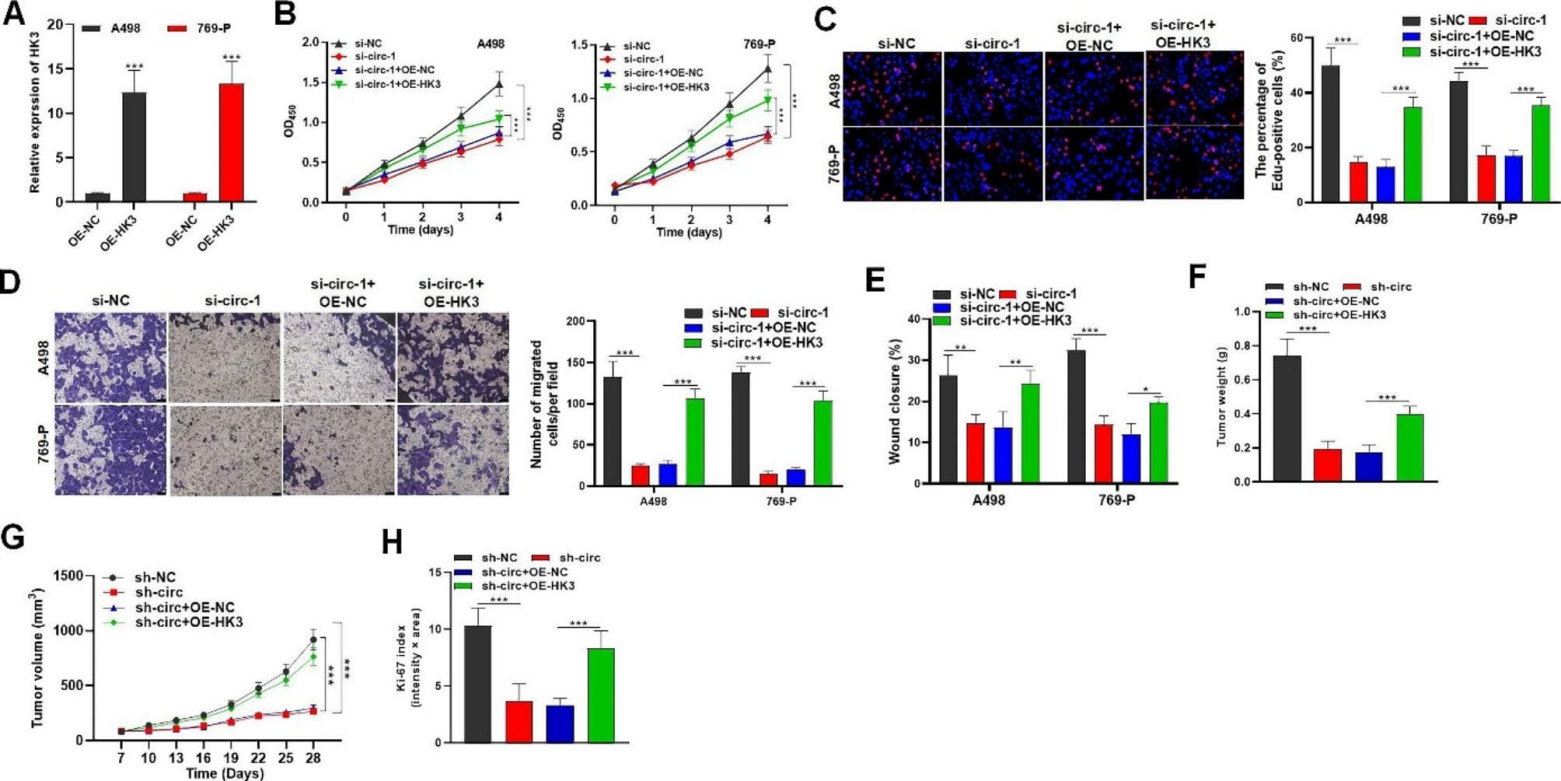
CircZBTB44 facilitated RCC growth and metastasis by up-regulating HK3. (**A**) HK3 overexpression efficiency in RCC cells was evaluated using qRT-PCR. (**B**) CCK-8 and (**C**) EdU assays were conducted to detect the viability and proliferation of RCC cells in indicated groups. (**D**) Transwell and (**E**) Wound healing assays were used to detect RCC cell migration in each group. (**F**) Mouse tumor weight and (**G**) volume in indicated transfection groups. (**H**) Ki67 protein expression in mouse tumor tissues of indicated groups was examined using IHC assays. *P < 0.05, **P < 0.01, ***P < 0.001

Rescue assays were performed to examine whether HK3 played a role in the regulation of circZBTB44 on RCC progression. HK3 was overexpressed using pcDNA3.1/HK3 vectors, and the transfection efficiency was confirmed by qRT-PCR (Fig. 7A). We found that circZBTB44 induced decrease in RCC cell viability and ratio of EdU-positive cells was significantly rescued after HK3 overexpression (Fig. 7B-C). Furthermore, the migrated RCC cell number and wound healing distance of RCC cells were reduced after silencing circZBTB44, which was revealed to be counteract by HK3 up-regulation (Fig. 7D-E). Additionally, the circZBTB44 deficiency induced decrease in xenograft tumor weight, volume and growth rate was significantly reversed by HK3 overexpression (Fig. 7F-G). Ki67 protein expression was reduced in tumor tissues of mice in the circZBTB44 knockdown groups, and was elevated by HK3 overexpression compared with the sh-circ + oe-NC groups (Fig. 7H).

### CircZBTB44 promoted M2 polarization of macrophages by up-regulating HK3

**Fig. 8:**
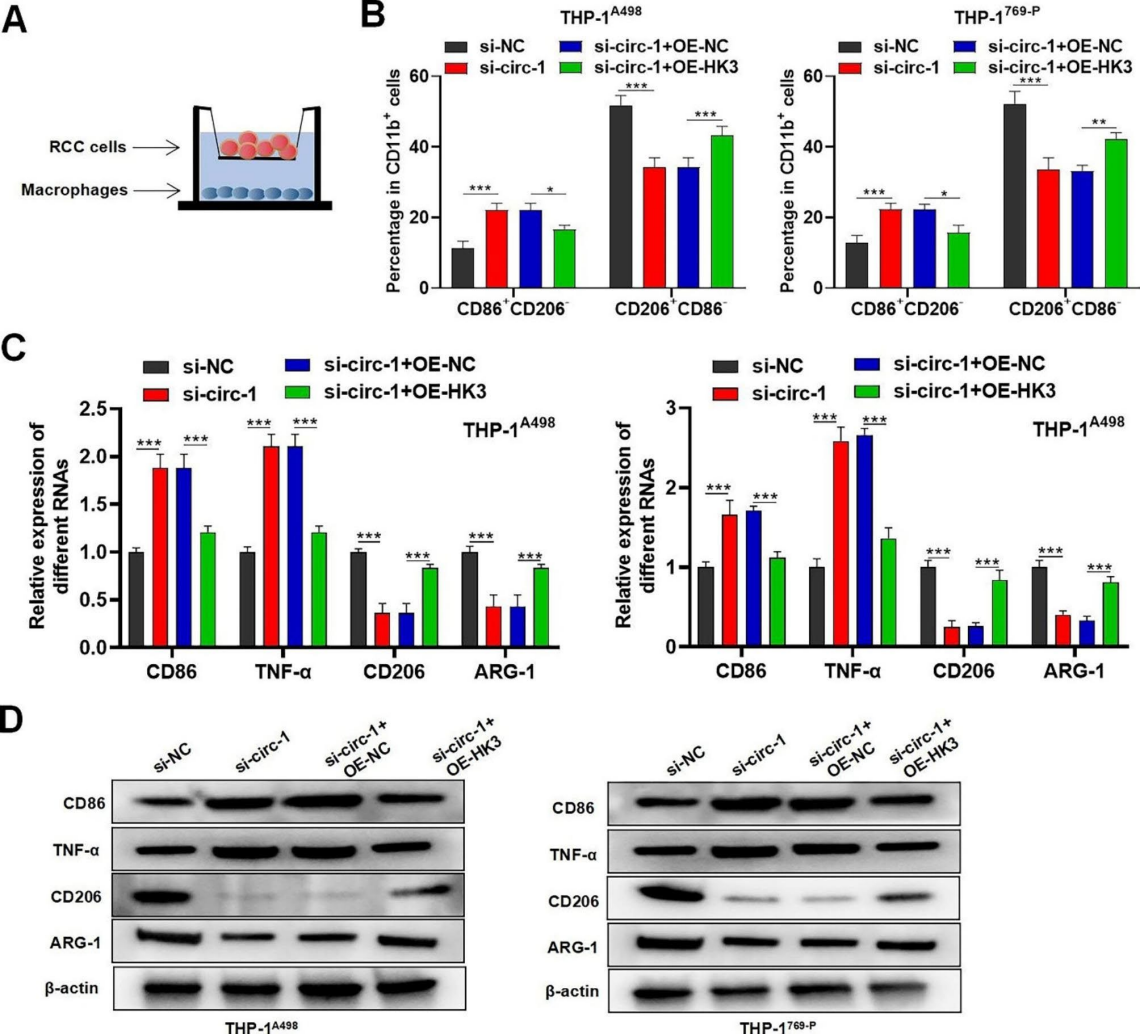
CircZBTB44 promoted M2 polarization of macrophages by up-regulating HK3. (**A**) The co-culture system of the M0 macrophages with RCC cells. (**B**) Flow cytometry was applied to examine the ratio of CD86^+^CD206^−^ macrophages and CD206^+^CD86^−^ macrophages. (**C**) qRT-PCR and (**D**) Western blot were performed to evaluate the mRNA and protein expression of M1-assocaited genes (CD86, TNF-α) and M2-associated genes (CD206, ARG-1) in macrophages co-cultured with transfected RCC cells. *P < 0.05, **P < 0.01, ***P < 0.001

A previous study has suggested that HK3 promoted the immune escape of RCC cells by stimulating the abundance of surface markers of infiltrating monocytes or macrophages [26]. Thus, we explored whether circZBTB44 regulated the M2 polarization of monocytes or macrophages by regulating HK3. TAMs were induced by co-culturing the M0 macrophages with RCC cells (Fig. 8A). As revealed by flow cytometry, circZBTB44 silencing significantly reduced the ratio of CD86^+^CD206^−^ macrophages and elevated the ratio of CD206^+^CD86^−^ macrophages, which was reversed by HK3 overexpression (Fig. 8B). Then we detected the expression of M1-assocaited genes (CD86, TNF-α) and M2-associated genes (CD206, ARG-1) in macrophages co-cultured with RCC cells. The results showed that circZBTB44 silencing decreased the expression of CD206 and ARG-1 and increased the levels of CD86 and TNF-α, which was significantly rescued by HK3 overexpression (Fig. 8C-D).

## Discussion

In this study, the upregulation of circZBTB44 in RCC tissues and cells was confirmed. CircZBTB44 was revealed to promote RCC cell malignancy and tumorigenesis via interaction with IGF2BP3 to up-regulate the expression of HK3. Additionally, circZBTB44 promoted M2 polarization of macrophages by up-regulating HK3. The findings of this study deepen our understanding on the regulatory mechanism of RCC.

m6A is critically implicated in the RNA metabolism process and is revealed to regulate the translation, degradation, splicing, translocation and folding of RNAs [35]. It plays an important role in diverse biological processes, including RNA-protein interaction [36, 37]. HNRNPC is an RNA binding protein that has been reported to regulate m6A modification in tumor development. For example, HNRNPC silencing suppressed pancreatic ductal adenocarcinoma growth and metastasis by modulating alternative splicing events [38]. In this study, we found that HNRNPC bound to circZBTB44 and regulated the m6A modification of circZBTB44 in RCC cells. Moreover, we revealed that HNRNPC promoted the translocation of circZBTB44 from nucleus to cytoplasm, facilitating the binding between circZBTB44 and IGF2BP3 in RCC cells. Moreover, IGFL2-AS1 binds to HNRNPC via m6A modification to regulate TP53INP2 expression in RCC cells, which enhances the sunitinib resistance of RCC cells [39]. Sunitinib can block the ATP-binding cassette (ABC) transporters [40]. The HNRNP family members can regulate ABC transporters [41, 42]. Whether HNRNPC regulates ABC transporters and whether Sunitinib blocks ABC transporters in an HNRNPC-dependent manner in RCC deserve further investigation.

IGF2BP3 is a member of the RBP family IGF2BPs, which interacted with downstream mRNAs and modulate the mRNA stability [34, 43]. The aberrant expression of IGF2BP3 is identified in various malignancies [43, 44]. A study has revealed that IGF2BP3 stabilized CDKN2B-AS1 to promote the progression of renal clear cell carcinoma via epigenetically activating NUF2 transcription [45]. In this study, we identified the interaction of IGF2BP3 and HK3. IGF2BP3 knockdown was revealed to significantly reduce the stability of HK3 in RCC cells. CircZBTB44, IGF2BP3 and HK3 mRNA were confirmed to be colocalized in cytoplasm, and circZBTB44 enhanced the IGF2BP3-mediated stabilization on HK3 mRNA. HK3 belongs to the hexokinase (HK) family and is suggested playing oncogenic roles in various cancers [46], and promotes proliferation and decreases apoptosis of clear cell renal cell carcinoma cells [26]. In our study, HK3 deficiency was demonstrated to inhibit the viability, proliferation potential and migration of RCC cells in vitro, and suppress xenograft tumorigenesis in vivo. Rescue assays revealed that circZBTB44 promoted RCC malignancy and M2 polarization of macrophages by up-regulating HK3. In addition, the solute carrier (SLC) transporters are promising targets for the treatment of cancer [47], including RCC [48]. Considering the metabolic gatekeeper role of SLC transporters [49] and the significant involvement of HK3 in metabolic reprograming, we speculate that HK3 regulation and/or function is also related with certain SLC transporters in RCC.

In conclusion, circZBTB44 is upregulated in RCC cells. It facilitates RCC cell proliferation and migration in vitro and promotes tumor growth in vivo by up-regulating HK3. HNRNPC mediates the interaction between circZBTB44 and IGF2BP3 via m6A modification, and circZBTB44 recruits IGF2BP3 to enhance the mRNA stability of HK3 (Supplementary file 1). The findings of this study deepen our understanding on the mechanism of RCC development and provide promising therapeutic targets for RCC treatment using new small molecule “silencers” to target circZBTB44.

## Electronic supplementary material

Below is the link to the electronic supplementary material.

Supplementary Material 1

## Data Availability

All materials underlying this study are available from the corresponding author based on a material transfer agreement.

## Abbreviations

RCC: renal cell carcinoma
ZBTB44: Zinc Finger and BTB Domain Containing 44
IGF2BP3: Insulin-like growth factor 2 mRNA-binding protein 3
HNRNPC: Heterogeneous Nuclear Ribonucleoprotein C
m^6^A: N6-methyladenosine
CircRNA: circular RNA
UTR: untranslated region
HK: hexokinase
TNF-α: tumor necrosis factor alpha
ARG1: Argininase-1
GAPDH: glyceraldehyde-3-phosphate dehydrogenase
RBP: RNA-binding protein
qRT-PCR: quantitative reverse transcription polymerase chain reaction
CCK-8: Cell-Counting kit-8
FISH: fluorescence in situ hybridization
EdU: 5-ethynyl-2’-deoxyuridine
IHC: immunohistochemistry
RIP: RNA immunoprecipitation
meRIP: methylated RNA immunoprecipitation
GST: glutathione-S-transferase
