# Supplementary figures and images for: CircZBTB44 promotes renal carcinoma progression by stabilizing HK3 mRNA structure
Source: Mol Cancer. 2023 Apr 27;22:77. doi: 10.1186/s12943-023-01771-5 (PMC10134651; doi:10.1186/s12943-023-01771-5)

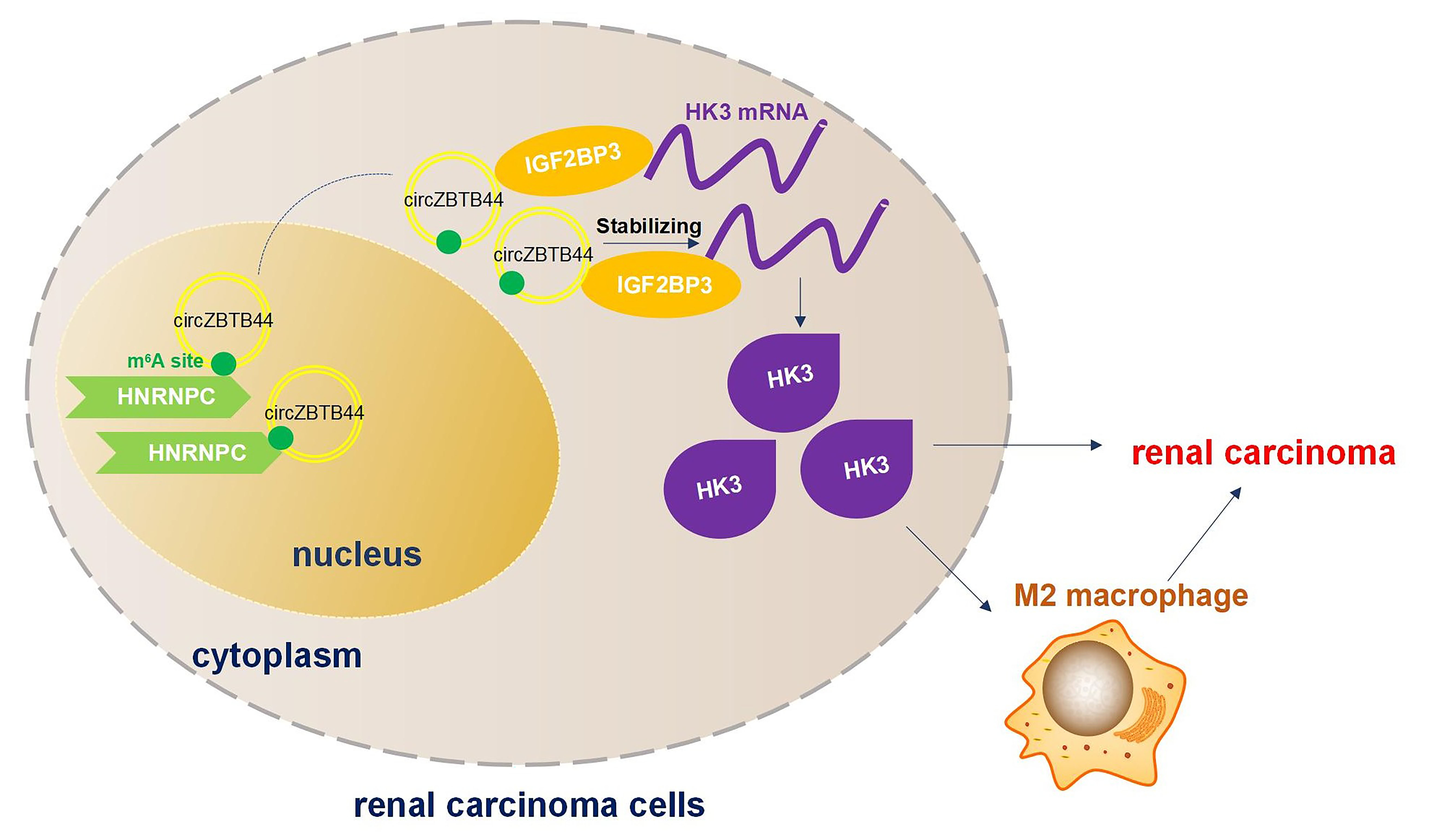

Supplement: Supplementary file 1 — Supplementary Material 1 [file 12943_2023_1771_MOESM1_ESM.jpg]
